# Identification of oxidative stress-related genes and potential mechanisms in atherosclerosis

**DOI:** 10.3389/fgene.2022.998954

**Published:** 2023-01-04

**Authors:** Chao Tang, Lingchen Deng, Qiang Luo, Guijun He

**Affiliations:** ^1^ Department of Cardiology, The Third People’s Hospital of Chengdu, The Affiliated Hospital of Southwest Jiaotong University, Chengdu, China; ^2^ College of Basic Medicine, Chengdu University of Traditional Chinese Medicine, Chengdu, China

**Keywords:** oxidative stress, atherosclerosis, GEO, LASSO, immune

## Abstract

Atherosclerosis (AS) is the main cause of death in individuals with cardiovascular and cerebrovascular diseases. A growing body of evidence suggests that oxidative stress plays an essential role in Atherosclerosis pathology. The aim of this study was to determine genetic mechanisms associated with Atherosclerosis and oxidative stress, as well as to construct a diagnostic model and to investigate its immune microenvironment. Seventeen oxidative stress-related genes were identified. A four-gene diagnostic model was constructed using the least absolute shrinkage and selection operator (LASSO) algorithm based on these 17 genes. The area under the Receiver Operating Characteristic (ROC) curve (AUC) was 0.967. Based on the GO analysis, cell-substrate adherens junction and focal adhesion were the most enriched terms. KEGG analysis revealed that these overlapping genes were enriched in pathways associated with Alzheimer’s disease and Parkinson’s disease, as well as with prion disease pathways and ribosomes. Immune cell infiltration correlation analysis showed that the immune cells with significant differences were CD4 memory activated T cells and follicular helper T cells in the GSE43292 dataset and CD4 naïve T cells and CD4 memory resting T cells in the GSE57691 dataset. We identified 17 hub genes that were closely associated with oxidative stress in AS and constructed a four-gene (aldehyde dehydrogenase six family member A1 (*ALDH6A1*), eukaryotic elongation factor 2 kinase (*EEF2K*), glutaredoxin (*GLRX*) and l-lactate dehydrogenase B (*LDHB*)) diagnostic model with good accuracy. The four-gene diagnostic model was also found to have good discriminatory efficacy for the immune cell infiltration microenvironment of AS. Overall, these findings provide valuable information and directions for future research into Atherosclerosis diagnosis and aid in the discovery of biological mechanisms underlying AS with oxidative stress.

## Introduction

Atherosclerosis (AS) is the main cause of death in individuals with cardiovascular and cerebrovascular diseases. Approximately 19 million deaths were estimated to be attributed to cardiovascular disease globally in 2020, with an increase of 18.7% from 2007 (Tsao et al., 2022). Several studies have demonstrated that oxidative stress plays a significant role in the pathophysiology of atherosclerosis (Kattoor et al., 2017; Khosravi et al., 2019; Yang et al., 2017). However, the biological mechanisms underlying AS with oxidative stress remain unclear.

Oxidative stress occurs when there is an imbalance favoring the increased generation of reactive oxygen species (ROS) or suppression of the antioxidant defense system in the body (Elahi et al., 2009). ROS are involved in inflammatory responses, apoptosis, cell growth, and alterations in the blood vessel tone. The production of ROS in vessel walls is increased under all conditions considered risk factors for atherosclerotic cardiovascular disease, including hypertension, diabetes, obesity and dyslipidemia (Bryk et al., 2017). Increasingly, research has shown that oxidative stress plays an important role in AS (Ridker et al., 2018; Tibaut et al., 2018; Libby, 2021). Atherosclerosis is now thought to be caused by inflammation in addition to dyslipidemia and other risk factors. As an example, C-reactive protein, a biomarker of inflammation, is demonstrated to be correlated with remnant lipoprotein concentrations (Ridker, 2016; Hansen et al., 2019). The role of inflammation in hypertension is also well established (Xiao and Harrison, 2020). However, the underlying mechanisms of genetic and epigenetics factors in the pathogenesis of atherosclerosis caused by oxidative stress is still unclear. As of now, genome-wide association studies have identified more than 50 independent loci associated with cardiovascular diseases that collectively account for 13.3% of their heritability (Nikpay et al., 2015). One study investigated the role of oxidative stress *ROM O 1* gene polymorphism and showed that subjects carrying the C allele were at a more than three folds increased risk of developing cardiovascular diseases (Tibaut et al., 2020). Therefore, an in-depth exploration of oxidative stress will help us understand how this disease develops and will also provide new ideas for its prevention and treatment.

Currently, studies on oxidative stress and AS focus on two topics; the first is the role of oxidative stress in the pathological process of AS, analyzing how oxidative stress is involved in AS (Yin et al., 2022) and the second is whether oxidative stress can be effectively targeted as a treatment for AS, specifically to slow down its progression, through clinical trials (Smolyaninov et al., 2022; Xu et al., 2022; Zhao et al., 2022). The purpose of this study was to examine the association between oxidative stress-related genes and AS at the gene level, as well as to construct a diagnostic model and to investigate its immune microenvironment.

## Materials and methods

### Acquisition of data and analysis of variance

**FIGURE 1:**
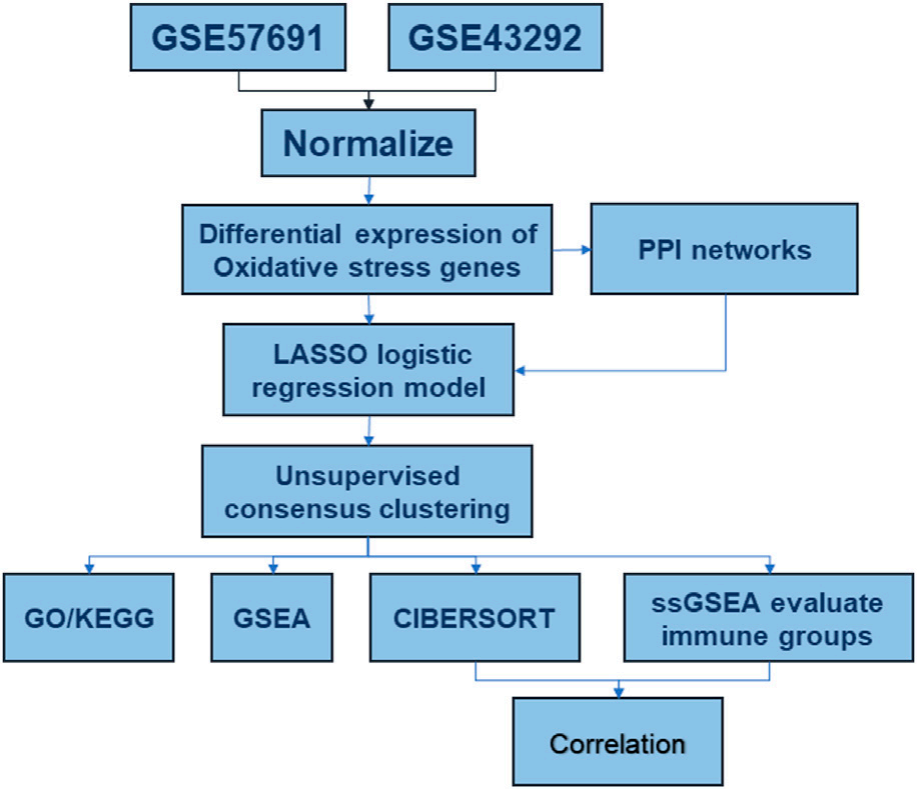
Analysis flow chart.

Two microarray datasets (GSE57691 (Biros et al., 2015) and GSE43292 (Ayari and Bricca, 2013) related to AS were obtained from the Gene Expression Omnibus (GEO) (http://www.ncbi.nlm.nih.gov/geo/). The GSE57691 and GSE43292 platforms were derived from GPL10558 and GPL6244, respectively. In total, 32 samples from patients with AS and 32 controls from GSE43292 and 58 samples from patients with AS and 10 controls from GSE57691 were analyzed in this study. The flow diagram of the study is shown in Figure 1.

### Acquisition of oxidative stress gene-related genes

The GeneCards database (Safran et al., 2010) provides comprehensive annotation information on human genes. We collected the oxidative stress genes from the GeneCards database (https://www.genecards.org/).

### Analysis of DEGs of oxidative stress genes

We used the R package “limma” to perform differential gene analysis between normal and disease samples in the datasets. To analyze the expression of genes encoding oxidative stress-related genes in all samples, we first used the R package pheatmap to plot a heat map of the expression of these genes in all samples and then grouped box plots based on two samples, namely normal and those from patients with AS.

### Correlation analysis between genes

To further dissect the correlation between the expression of related genes in all patients with AS, Pearson correlation values between two genes were calculated, and correlation coefficients with an absolute value greater than 0.5 and a *p*-value <0.05 were considered correlated. Scatter plots of correlations between eligible gene pairs were plotted, and correlation curves were fitted using the R package ggplot2.

### Construction of the diagnostic model

LASSO regression (Vasquez et al., 2016) was used to select features, reduce dimensionality, and create disease profiles associated with genes related to oxidative stress. Columnar line plots were used to identify disease-associated risk factors. To validate the predictive efficacy of the diagnostic model, single-gene ROC curves were plotted using the R package pROC, and the AUC was calculated. To illustrate the validity of the nomogram, validation was performed using an in-house dataset and the decision curve analysis (DCA) curves.

### Construction of PPI network

To analyze protein interactions, PPIs were established and hub genes were identified using an online tool (Search Tools for Retrieval of Interacting Genes, STRING) (Mering et al., 2003). The PPI network was visualized using Cytoscape (Shannon et al., 2003) software. CytoHubba (Rongbin et al., 2019) was utilized to calculate three indicators to evaluate the importance of each node, and the top 10 nodes were chosen. Hub genes served as the common nodes. We then conducted further prediction studies on hub nodes based on the TarBase (Wang et al., 2013), miRecords (Fornes et al., 2020), and miRTarBase (Huang et al., 2020) databases for miRNAs and transcription factors of hub nodes. We used the ENCODE database (Wang et al., 2013), JASPAR database (Fornes et al., 2020), and the ChEA database (Lachmann et al., 2010) for prediction.

### Unsupervised clustering of samples

The R package “ConsensusClusterPlus” (Wilkerson and Hayes, 2010) was used to perform unsupervised clustering of samples based on oxidative stress gene regulators. The resampling method disrupts the original dataset such that each resampled sample is clustered, and then, the results of multiple clustering analyses are evaluated together to provide a consensus assessment.

### Functional enrichment analysis

We conducted Kyoto Encyclopedia of Gene and Genome (KEGG) (Kanehisa and Goto, 2000)and gene ontology (GO) (Ashburner et al., 2000) analyses to understand the functions of these hub genes. To determine the biological activities of the genes and linked pathways, the clusterProfiler (Wilkerson and Hayes, 2010) R package was used to conduct an enrichment analysis. Using the reference gene set “c2. cp.kegg.v7.4. Entrez. gmt” from the MSigDB database (Liberzon et al., 2015), we compared the biological processes between the two samples and used the GSEA (Subramanian et al., 2005) and Gene Set Variation Analysis (GSVA) (Hänzelmann et al., 2013) methods included in the R package “clusterProfiler” for enrichment analysis and visualization of the dataset.

### Immune infiltration analysis

To compare the levels of immune cell infiltration between the two groups of samples, immune cell infiltration was assessed using the R package GSVA based on ssGSEA (Subramanian et al., 2005). To maximize the accuracy of the results, the level of immune cell infiltration was assessed using the R package CIBERSORT (Steen et al., 2020).

### Statistical analysis

All data processing and analyses were performed using R software (version 4.0.2). Student’s paired t-tests were used to analyze data when comparing the two groups, and *p* < 0.05 was considered statistically significant.

## Results

### Data preprocessing and identification of differentially expressed genes (DEGs)

**FIGURE 2:**
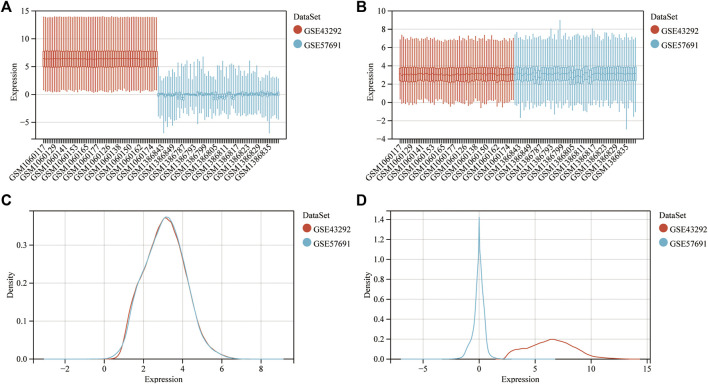
Dataset merging, standardization and normalization. **(A)** Box plots of the expression spectrum matrix of GSE57691 and GSE43292 data sets before calibration; **(B)** Box plots of the expression spectrum matrix of GSE57691 and GSE43292 data sets after calibration; **(C)** Density plots of the expression spectrum matrix of GSE57691 and GSE43292 data sets before calibration; **(D)** Density plots of the expression spectrum matrix of GSE57691 and GSE43292 data sets after calibration.

In Figures 2A–D, the expression distribution characteristics of the two datasets before and after data pretreatment are presented. Almost all median values were on a straight line, proving that they were normalized. Among the DEGs, the upregulated and downregulated genes were identified with a False Discovery Rate <0.05 and a |log2FC| > 0.58. After data processing, 758 DEGs were identified, with 515 upregulated and 243 downregulated genes. The information summary of the two datasets was showed in Table 1.

**TABLE 1:** Dataset information summary.

Dataset	Normal	Atherosclerosis	Platform	Organism	Tissue
GSE43292	32	32	GPL6244	Human	atheroma plaque
GSE57691	10	58	GPL10558		full thickness aortic wall

**FIGURE 3:**
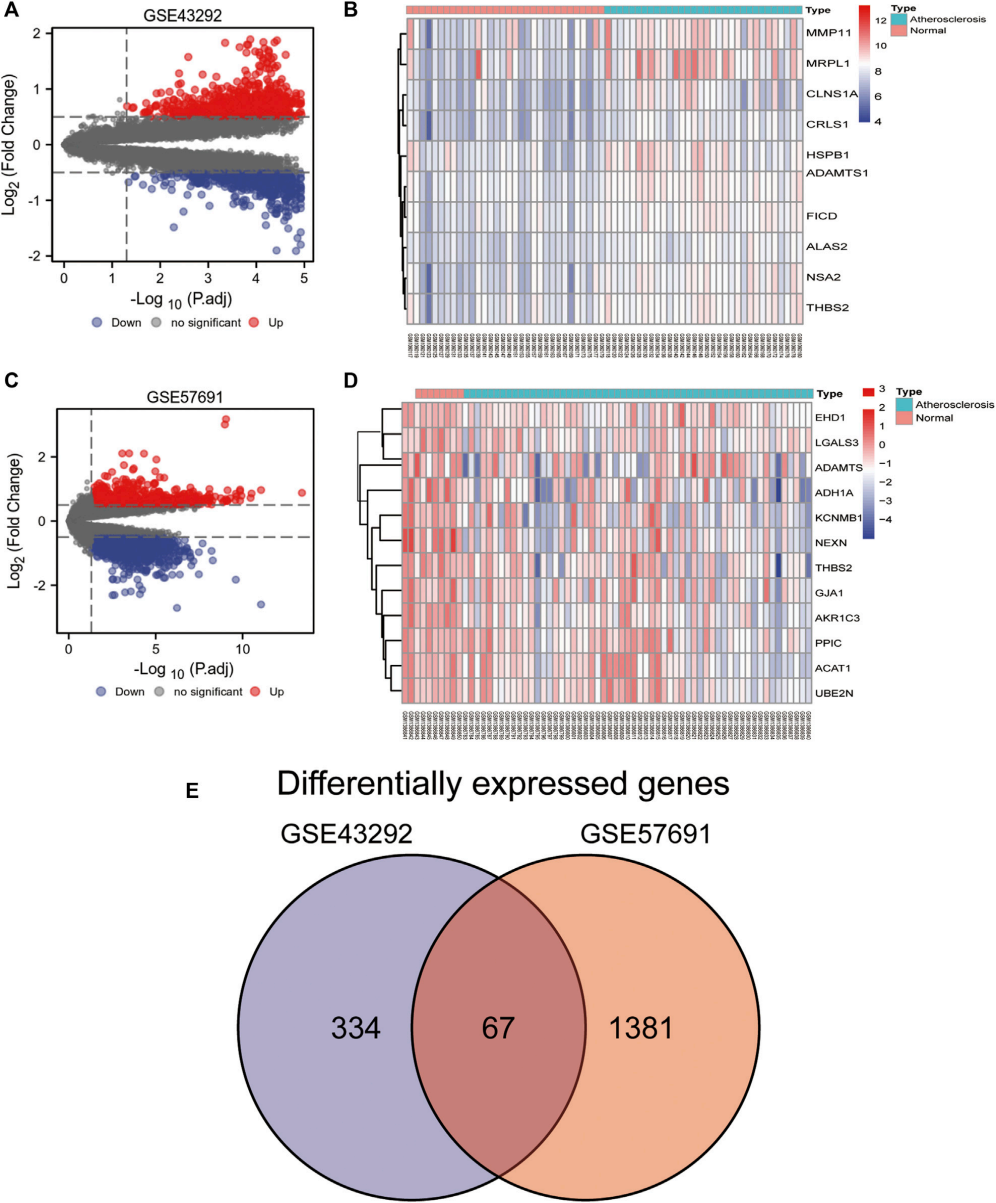
Differential expression of oxidative stress-related genes. **(A,B)** Volcano plot of DEGs in GSE43292 and GSE57691; **(C,D)** Heat map of DEGs in GSE43292 and GSE57691; **(E)** Venn diagram of DEGs in the two datasets.

We analyzed the differences in the expression of oxidative stress-related genes between normal and diseased samples in the GSE43292 and GSE57691 datasets, which are presented as volcano plots (Figures 3A–C) and heat maps (Figures 3B–D). In GSE43292, the expression of 221 differential genes was upregulated and that of 181 was downregulated. The DEGs in GSE57691 included 231 upregulated and 1,219 downregulated genes. A Venn diagram showing DEGs in GSE57691 and GSE43292 is shown in Figure 3E. In total, 67 intersecting differential oxidative stress-related genes were obtained in the GSE57691 and GSE43292 datasets (Additional file 1).

### Construction of protein–protein interaction (PPI) network

**FIGURE 4:**
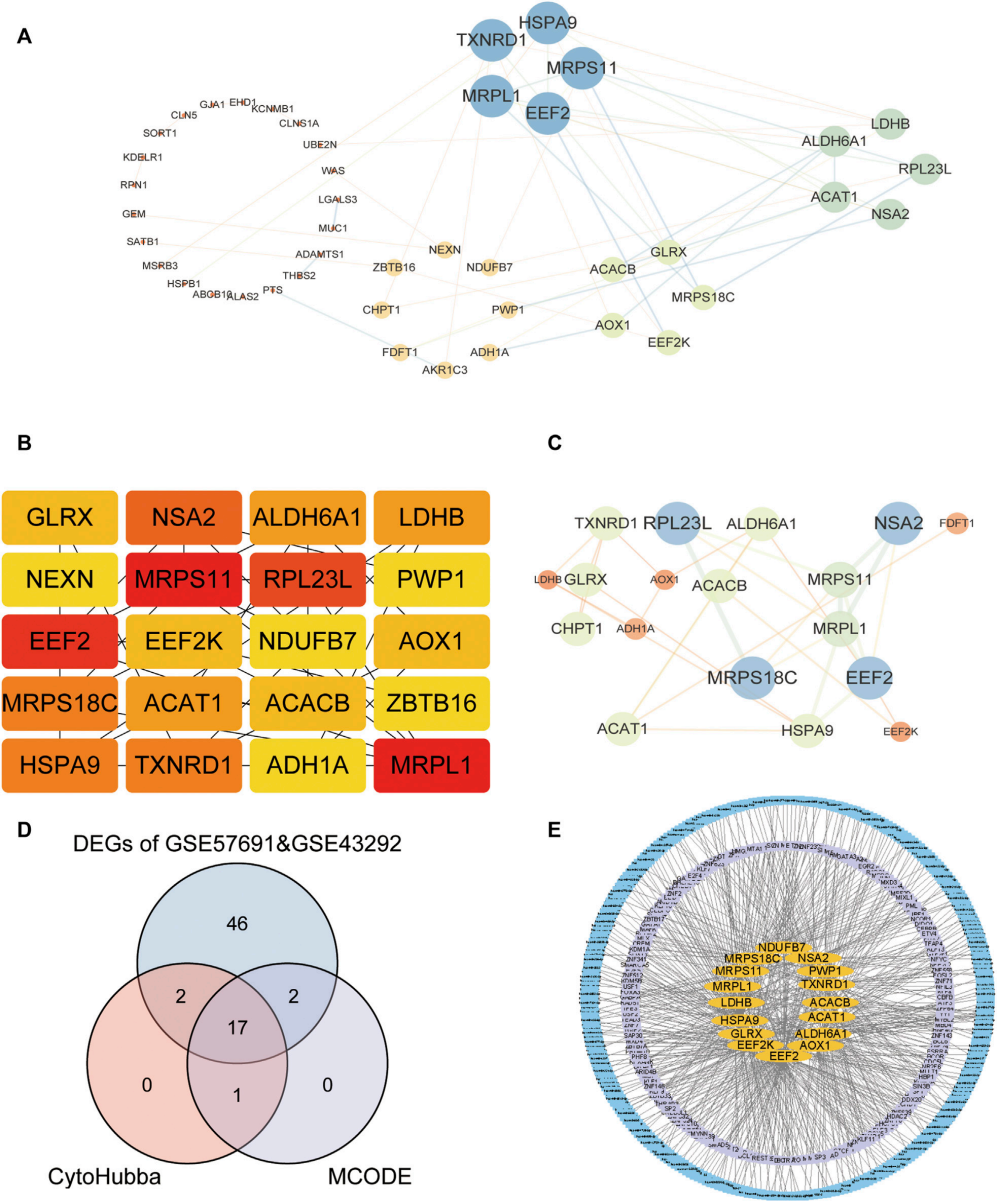
Construction of molecular interaction network of DEGs and screening of co-expressed hub gene. **(A)** STRING database for 67 intersection of DEGs; **(B)** CytoHubba plug-in screening of top-20 hub genes; **(C)** Cytoscape’s MCODE analysis of hub genes; **(D)** Venn diagram showing the intersection of two methods to obtain 17 closely related co-expressed genes; **(E)** TF and miRNA prediction of the 17-gene diagnostic model, the yellow color located in the middle represents the 17-gene oxidative stress-related diagnostic model, the middle purple layer represents its associated TF; the outer blue layer represents its targeted miRNA.

We first analyzed the interactions among 67 intersecting differentially expressed oxidative stress-related genes in the GSE57691 and GSE43292 datasets and constructed PPI networks of differentially expressed oxidative stress-related genes for the 67 intersecting genes using the STRING database. The highest confidence interaction score was set to 0.4 (Figure 4A). The top 20 closely linked genes were screened using the CytoHubba plugin (Figures 4B, C). The intersection of the two approaches was demonstrated using a Venn diagram to identify 17 closely related differential oxidative stress-related genes (Figure 4D), including acetyl-CoA acetyltransferase 1 (*ACAT1*), acetyl-coenzyme A carboxylase beta (*ACACB*), alcohol dehydrogenase 1A (*ADH1A*), aldehyde dehydrogenase six family member A1 (*ALDH6A1*), alcohol oxidase 1 (*AOX1*), eukaryotic elongation factor 2 kinase (*EEF2*), eukaryotic elongation factor 2 kinase (*EEF2K*), glutaredoxin (*GLRX*), heat shock protein family A member 9 (*HSPA9*), l-lactate dehydrogenase B (*LDHB*), mitochondrial ribosomal protein L1 (*MRPL1*), mitochondrial ribosomal protein S11 (*MRPS11*), mitochondrial Ribosomal Protein S18C (*MRPS18C*), *NADH*: ubiquinone oxidoreductase subunit B7 (NDUFB7), nop seven-associated 2 (*NSA2*), periodic tryptophan protein 1 (*PWP1*) and thioredoxin reductase 1 (*TXNRD1*). We predicted 17 closely related differential oxidative stress-related miRNAs or transcription factor (TF) -regulated genes targeted by the diagnostic model. In total, 179 associated TF-regulated genes and 285 miRNAs were obtained, and the network was constructed (Figure 4E).

### Molecular subtype construction and analysis based on oxidative stress

**FIGURE 5:**
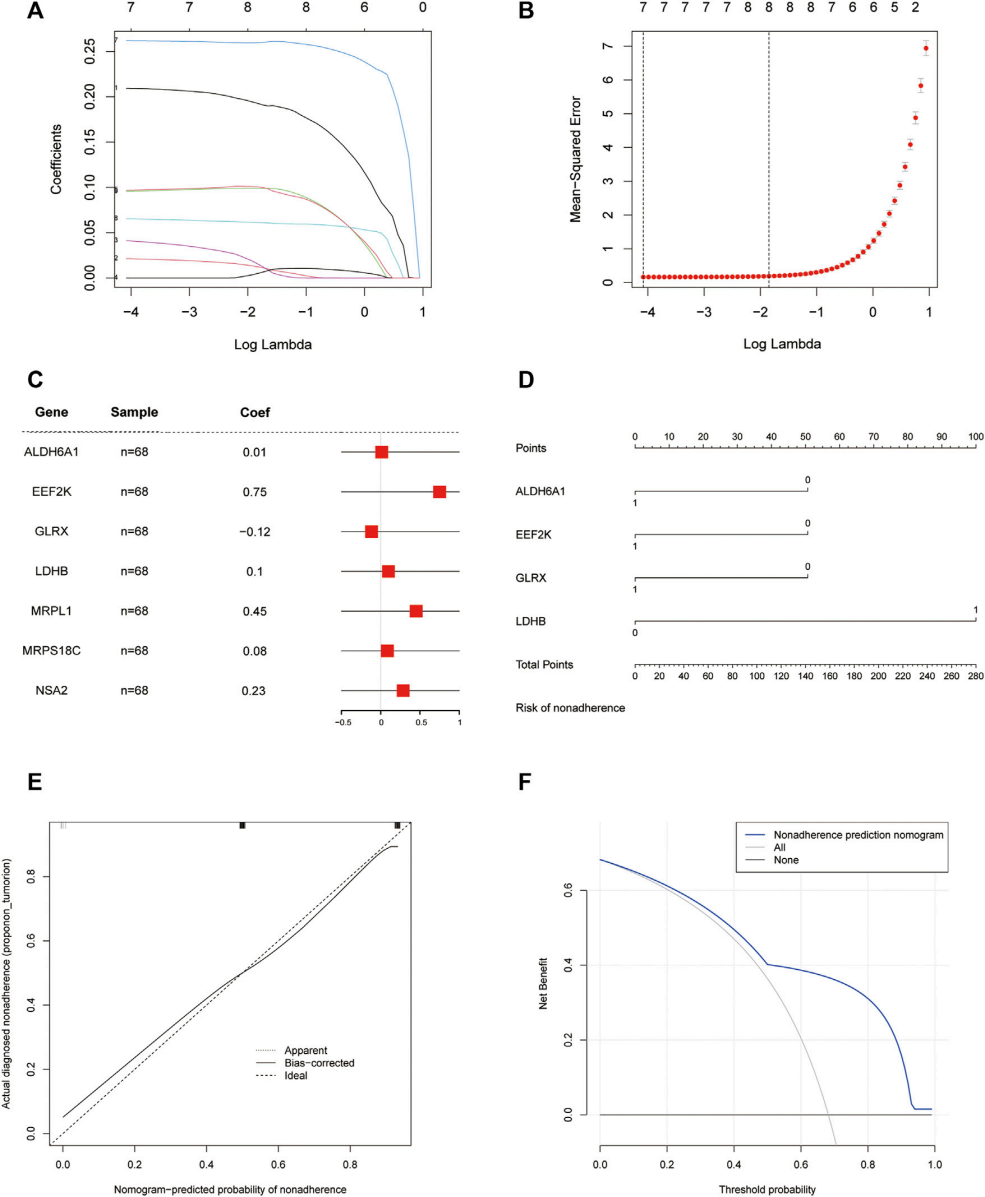
Diagnostic model. **(A)** LASSO logistic regression algorithm screening diagnostic marker lambda value visualization; **(B)** LASSO logistic regression algorithm screening diagnostic marker min value visualization; **(C)** Forest plot of seven meaningful oxidative stress-related arterial disease risk genes with significant biological significance; **(D)** Column line graph of a 4-gene diagnostic marker model composed of *ALDH6A1*, *EEF2K*, *GLRX*, and *LDHB*; **(E)** Calibration curve of non-correlated nomogram; **(F)** Decision curve analysis of the diagnostic model.

We used the least absolute shrinkage and selection operator (LASSO) algorithm for these 17 genes for further gene diagnostic model construction for closely related expression (Figures 5A, B).

We identified seven genes, *ALDH6A1*, *EEF2K*, *GLRX*, *LDHB*, *MRPL1*, *MRPS18C,* and *NSA2*, which are biologically significant for oxidative stress, in the risk forest plot of 17 genes (Figure 5C). For validation, column line plots of the atherosclerosis diagnostic biomarkers (oxidative stress-related genes) screened in the dataset were plotted to demonstrate the discriminatory efficacy of these predictive diagnostic markers for AS (Figure 5D). The calibration curves of the non-correlated nomogram predictions were further analyzed and plotted in the cohort (Figure 5E). Calibration curves showed that four genes (aldehyde dehydrogenase six family member A1 (*ALDH6A1*), eukaryotic elongation factor 2 kinase (*EEF2K*), glutaredoxin (*GLRX*) and l-lactate dehydrogenase B (*LDHB*)) could serve as diagnostic markers for AS.

### Validation of oxidative stress-related DEGs and diagnostic efficacy

**FIGURE 6:**
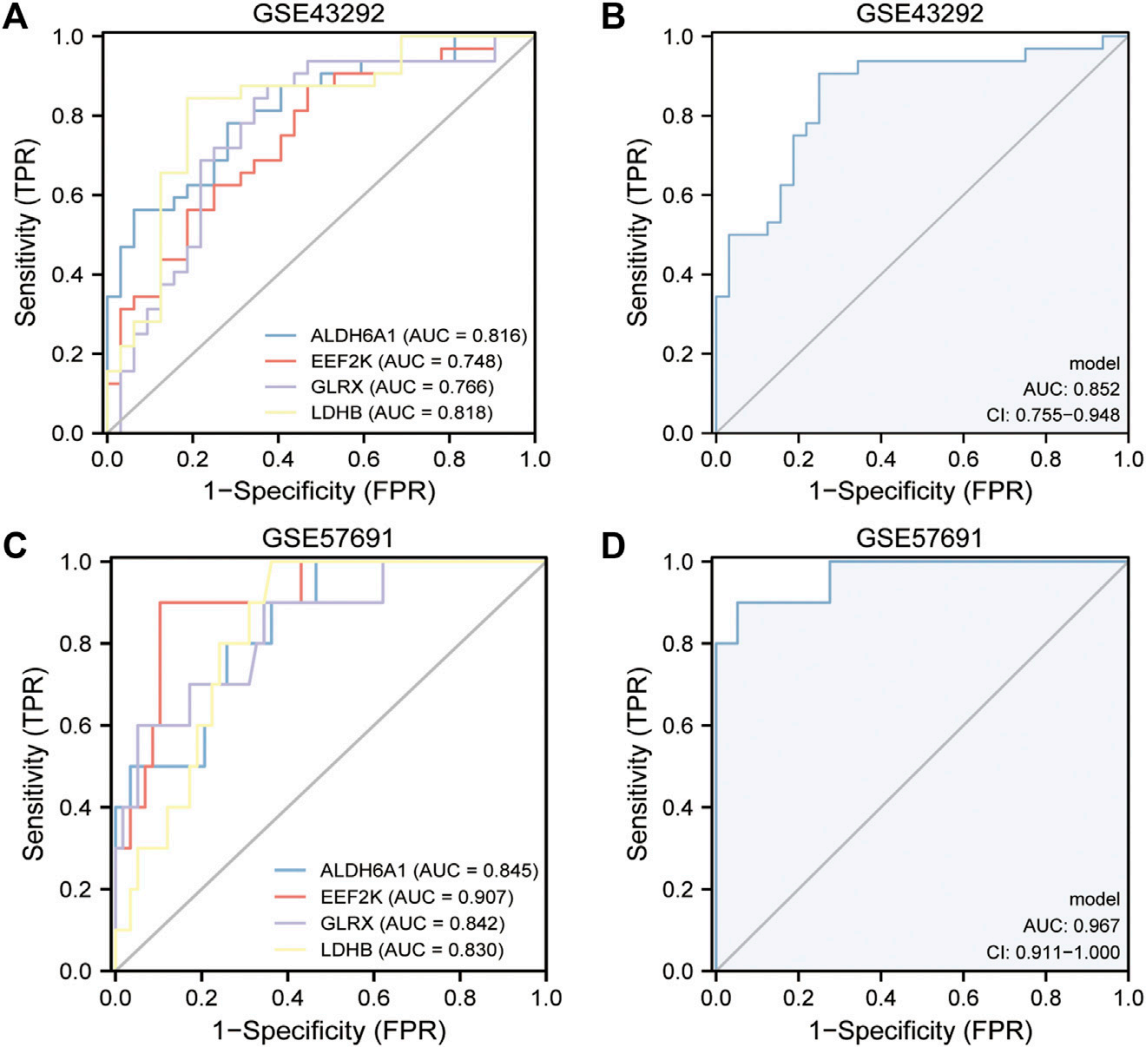
Validation of the diagnostic efficacy of 4-gene diagnostic model. **(A)** ROC curves of the diagnostic efficacy in the GSE43292 dataset (independent index); **(B)** ROC curves of the diagnostic efficacy in the GSE43292 dataset (joint index); **(C)** ROC curves of the diagnostic efficacy in the GSE57691 dataset (independent index); **(D)** ROC curves of the diagnostic efficacy in the GSE57691 dataset (joint index).

The area under the curve (AUC) values of the ROC curves were used to reflect the discriminatory efficacy, and an AUC >0.7 indicated high diagnostic efficacy. Figures 6A–C showed the independent expression of *ALDH6A1*, *EEF2K*, *GLRX*, and *LDHB* in the GSE43292 and GSE57691 datasets. The results revealed that the AUC of *ALDH6A1* equals 0.816, *EEF2K* equals 0.748, *GLRX* equals 0.766, and *LDHB* equals 0.818 in the GSE43292 dataset. The AUC values were also good in GSE57691 datasets. Figures 6B–D show the combined diagnostic efficacy of the expression of *ALDH6A1*, *EEF2K*, *GLRX*, and *LDHB* in GSE43292 and GSE57691 datasets (Figure 6B, AUC: 0.852, CI: 0.755–0.948 for the four-gene diagnostic model of the GSE43292 dataset; Figure 6D, AUC: 0.967, CI: 0.911–1.000 in GSE57691 datasets) based on the ROC curves.

**FIGURE 7:**
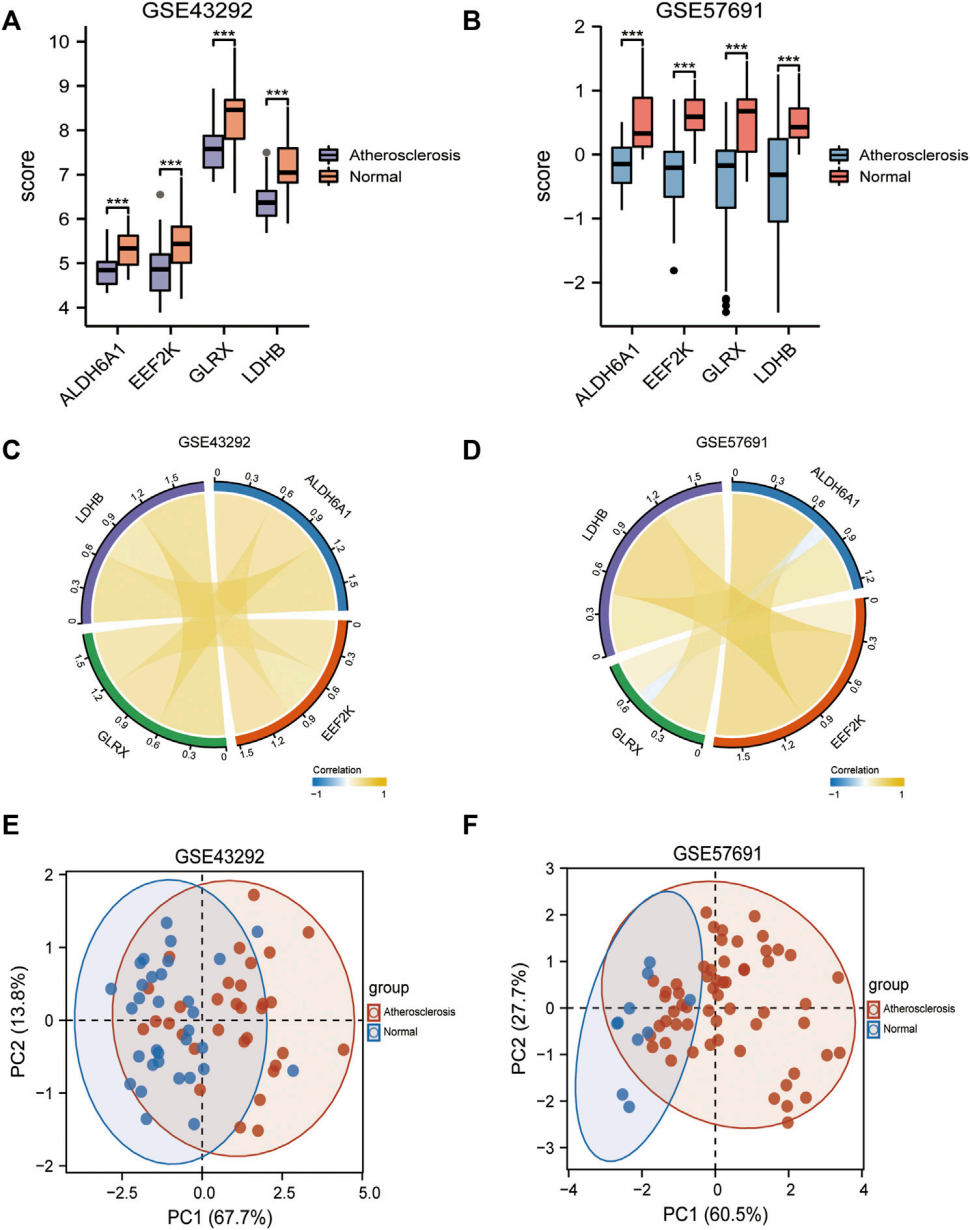
Differential expression in diagnostic models. **(A)** Box plots of differential expression of diagnostic models in the GSE43292; **(B)** Box plots of differential expression of diagnostic models in the GSE57691; **(C)** Correlation network plots between 4-gene diagnostic models in the GSE43292; **(D)** Correlation network plots between 4-gene diagnostic models in the GSE57691; **(E)** PCA clustering plot of differential oxidative stress genes between atherosclerosis and normal groups in the GSE43292 dataset; **(F)** PCA clustering plots. * is less than 0.05, ** is 0.01, *** is 0.001, **** is 0.0001, and no symbol means the difference is not significant.

Subsequently, we analyzed the differences in the expression of diagnostic models in the GSE43292, GSE57691 dataset, presented as box plots (Figures 7A, B), and Figures 7C, D shows the correlation analysis for the expression of the four genes in the atherosclerosis group. Figures 7E, F shows the differentiation of oxidative stress-related genes in the mRNA expression profile matrix of the GSE43292, GSE57691 dataset by PCA clustering plot for atherosclerotic and normal samples.

### Molecular typing analysis of four-gene diagnostic model

**FIGURE 8:**
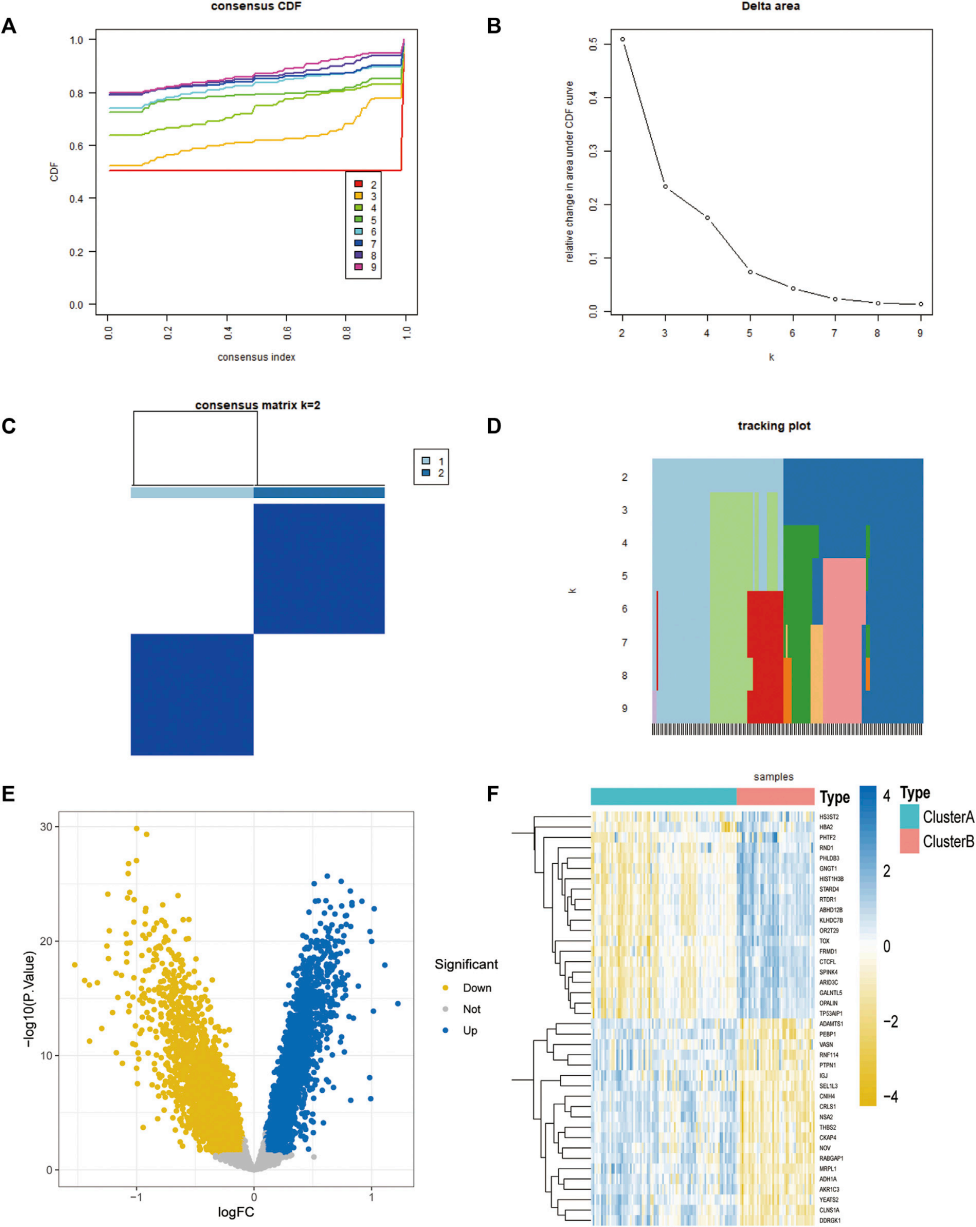
Molecular typing of the 4-gene diagnostic model of atherosclerosis associated with oxidative stress in GSE57691 vs. GSE43292. **(A)** CDF plot of consistent clustering; **(B)** Delta plot of consistent clustering, reflecting the optimal number of classifications; **(C)** Heat map of the difference between 2-classification clustering groups; **(D)** Fractal plot of consistent clustering samples **(E,F)** Consistent clustering grouping of 4-gene diagnostic model, Volcano plot and heat map.

Unsupervised consensus clustering was performed on the four-gene diagnostic model with GSE57691 and GSE43292. The expression of these genes was further assessed by cluster analysis of the samples in the two sets according to the different subclasses. The resulting clustering of samples within a class was more stable relative to the sampling variance and could represent a true subclass structure. The best k value of two was selected based on the delta plot. The grouped cases contained atherosclerotic samples classified as A (*n* = 64) and B (*n* = 68) (Figures 8A–D). Furthermore, we explored oxidative stress-related gene diagnostic models obtained using the training set LASSO regression algorithm (Figures 8E, F).

### Functional enrichment analysis

**FIGURE 9:**
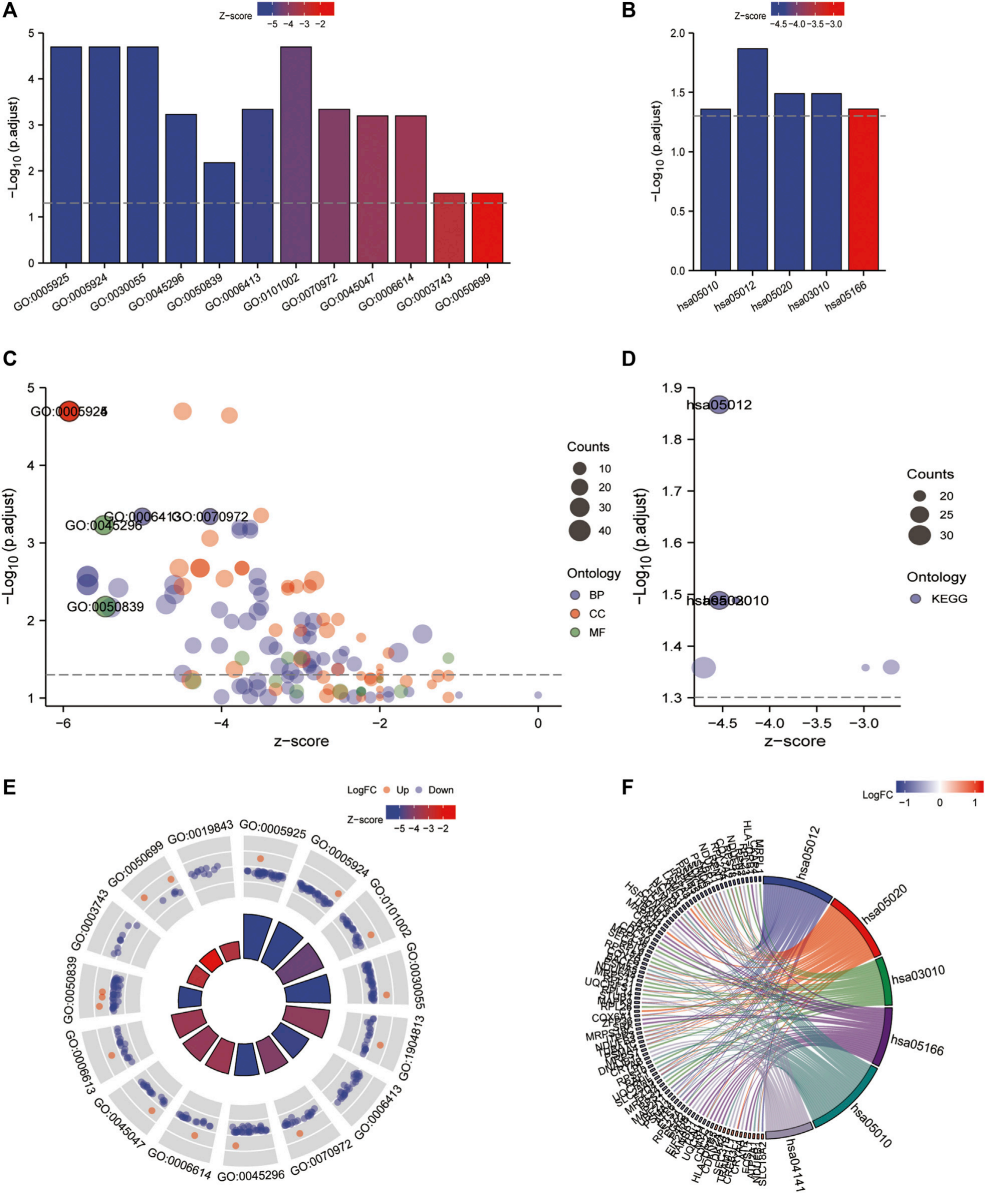
GO/KEGG enrichment analysis of the 4-gene diagnostic marker model in GSE57691 vs. GSE43292. **(A,B)** Bubble plot of GO/KEGG enrichment analysis; **(C,D)** Bar graph of GO/KEGG enrichment analysis; **(E,F)** String plot of GO enrichment analysis; circle plot of GO/KEGG enrichment analysis.

**FIGURE 10:**
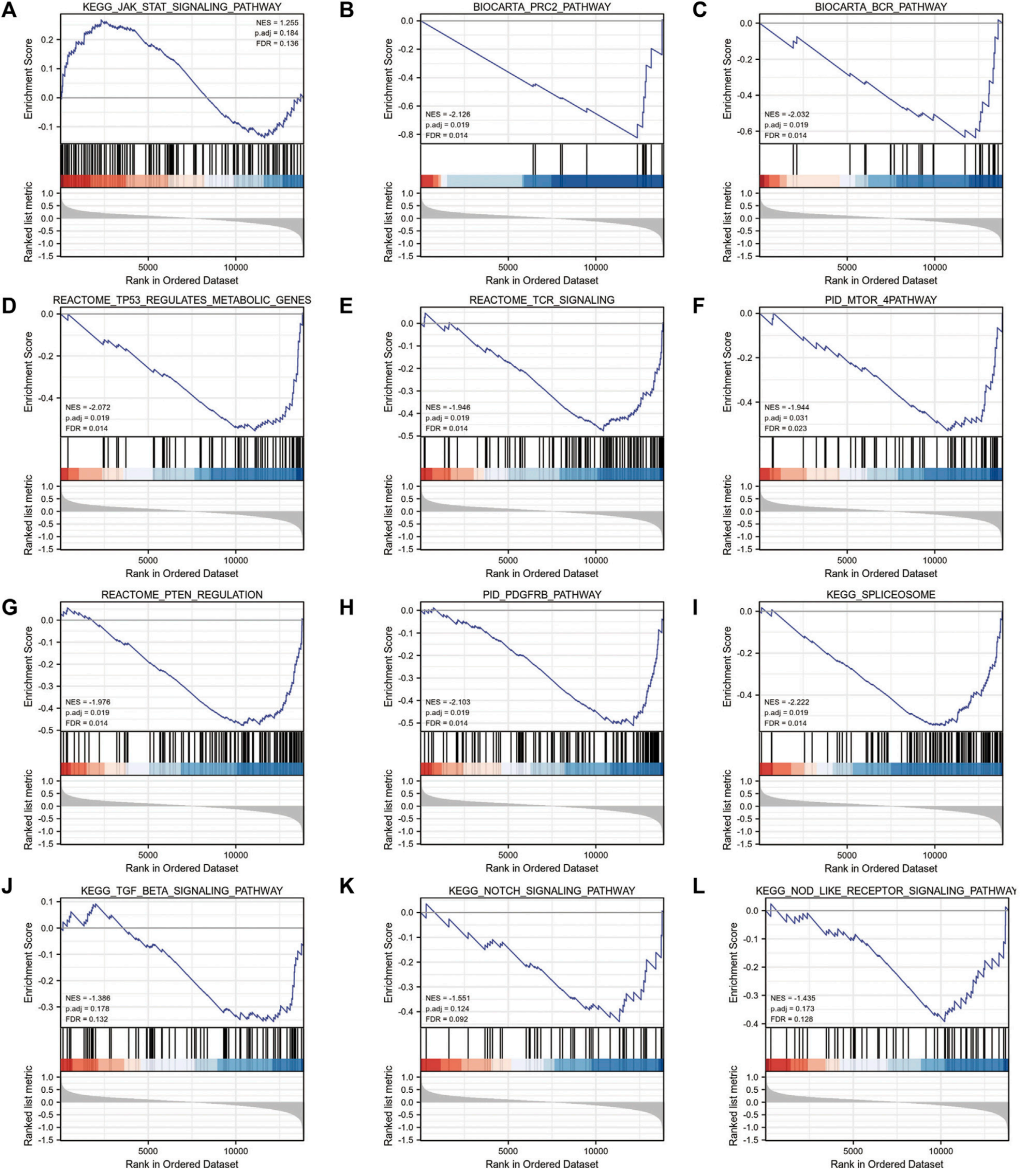
GSEA enrichment analysis of 4-gene diagnostic model. **(A)** KEGG_JAK_STAT_SIGNALING_PATHWAY; **(B)** BIOCARTA_PRC2_PATHWAY; **(C)** BIOCARTA_BCR_PATHWAY **(D)** REACTOME_TP53_REGULATES_METABOLIC_GENES; **(E)** REACTOME_TCR_SIGNALING; **(F)** PID_MTOR_4PATHWAY; **(G)** REACTOME_PTEN_REGULATION; **(H)** PID_PDGFRB_PATHWAY; **(I)** KEGG_SPLICEOSOME; **(J)** KEGG_TGF_BETA_SIGNALING_PATHWAY; **(K)** KEGG_NOTCH_SIGNALING_PATHWAY; **(L)** KEGG_NOD_LIKE_RECEPTOR_SIGNALING_PATHWAY.

Gene ontology (GO) (Ashburner M et al., 2000) enrichment results demonstrated that focal adhesion, cell-substrate adherens junction, cell-substrate junction, cadherin binding, et al. were significantly enriched (Table 2). Alzheimer’s disease, Parkinson’s disease, prion disease, ribosome, and human T cell leukemia virus one infection pathways were significantly enriched based on KEGG analysis (Figure 9 and Table 3). The Gene Set Enrichment Analysis (GSEA) results suggested that there were no significant differences in JAK/STAT, BCR, PRC2, MTOR 4, TCR, TGF β, TP53, Notch, and NOD-like receptor signaling pathways, but that spliceosome-related pathways were significantly enriched (Figure 10).

**TABLE 2:** Results of GO enrichment analysis of 106 co-expressed genes for 4-gene diagnostic marker model.

Ontology	Id	Description	p.Adjust	Qvalue
CC	GO:0005925	focal adhesion	2.02e-05	1.80e-05
CC	GO:0005924	cell-substrate adherens junction	2.02e-05	1.80e-05
CC	GO:0030055	cell-substrate junction	2.02e-05	1.80e-05
MF	GO:0045296	cadherin binding	5.92e-04	5.69e-04
MF	GO:0050839	cell adhesion molecule binding	0.007	0.006
BP	GO:0006413	translational initiation	4.59e-04	4.36e-04
CC	GO:0101002	ficolin-1-rich granule	2.02e-05	1.80e-05
BP	GO:0070972	protein localization to endoplasmic reticulum	4.59e-04	4.36e-04
BP	GO:0045047	protein targeting to ER	6.34e-04	6.02e-04
BP	GO:0006614	SRP-dependent cotranslational protein targeting to membrane	6.34e-04	6.02e-04
MF	GO:0003743	translation initiation factor activity	0.031	0.029
MF	GO:0050699	WW domain binding	0.031	0.029

**TABLE 3:** Results of KEGG enrichment analysis of 106 co-expressed genes for 4-gene diagnostic marker model.

Ontology	Id	Description	p.Adjust	Qvalue
KEGG	hsa05010	Alzheimer disease	0.044	0.042
KEGG	hsa05012	Parkinson disease	0.014	0.013
KEGG	hsa05020	Prion disease	0.032	0.031
KEGG	hsa03010	Ribosome	0.032	0.031
KEGG	hsa05166	Human T-cell leukemia virus 1 infection	0.044	0.042

### The ssGSEA enrichment analysis for immune cell infiltration

**FIGURE 11:**
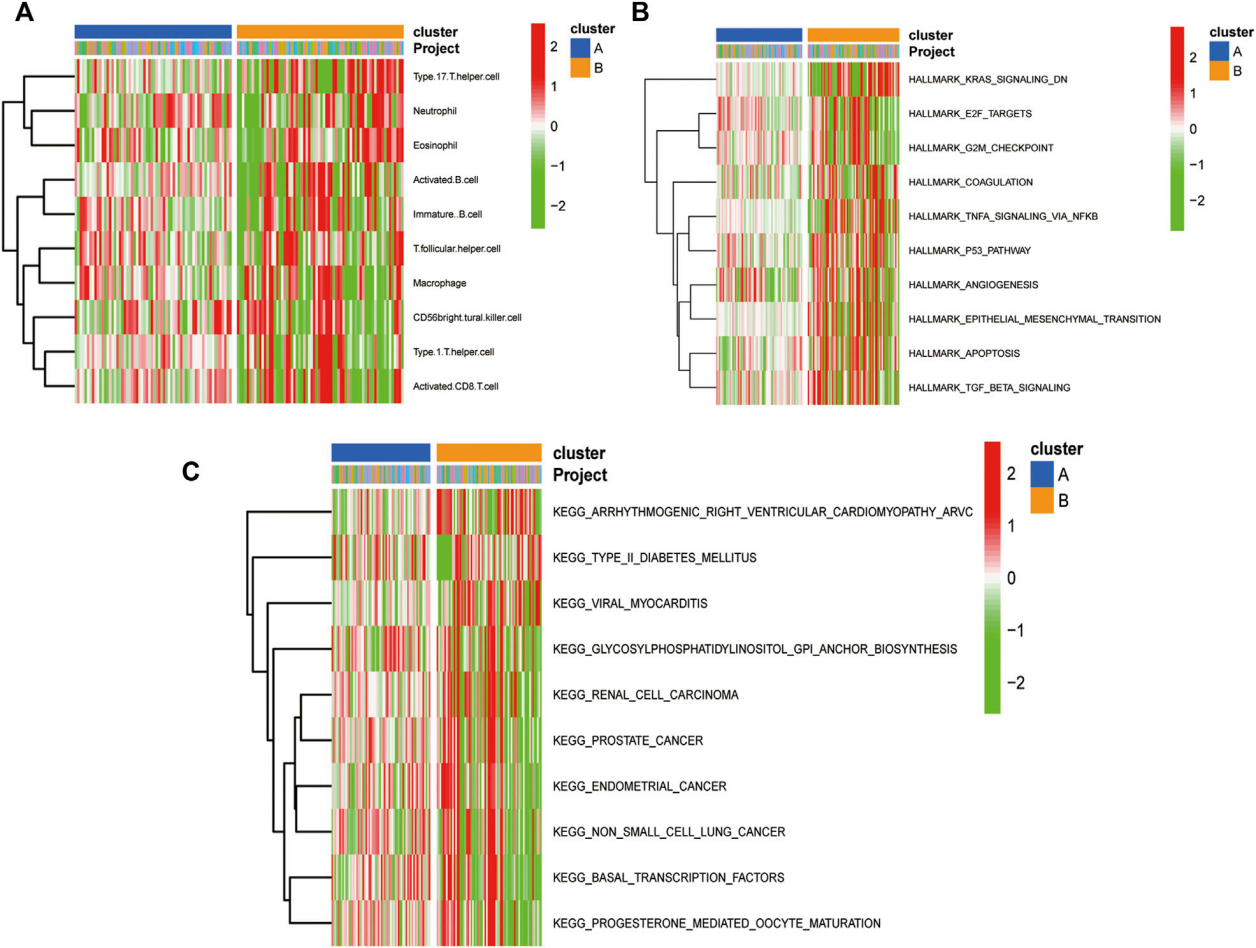
Molecular typing gsva enrichment analysis of consistent clustering of oxidative stress-related genes. **(A)** Heat map of inter-group differences in the ssGSEA immune gene set comparison between the consistent clustering subgroups; **(B)** Heat map of enrichment differences in the “h.all.v7.2. symbols.gmt” set; **(C)** Heat map of enrichment differences in the “c2. cp.v7.2. symbols.gmt” set between the consistent clustering subgroups.

The results of ssGSEA immune gene collection pathway enrichment analysis between the groups showed significant differences in the degree of infiltration (Figure 11A). The HALLMARK pooled clustering group showed significant differences between the two clustering groups in KRAS, TNFA/NFKB, P53, and TGFβ signaling pathways (Figure 11B). The KEGG pooled clustering group also showed significant differences between the two clustering groups in arrhythmia, ventricular cardiomyopathy, viral myocarditis, and other pathways (Figure 11C).

### Construction of immune signature subtypes and assessment of immune cell infiltration

**FIGURE 12:**
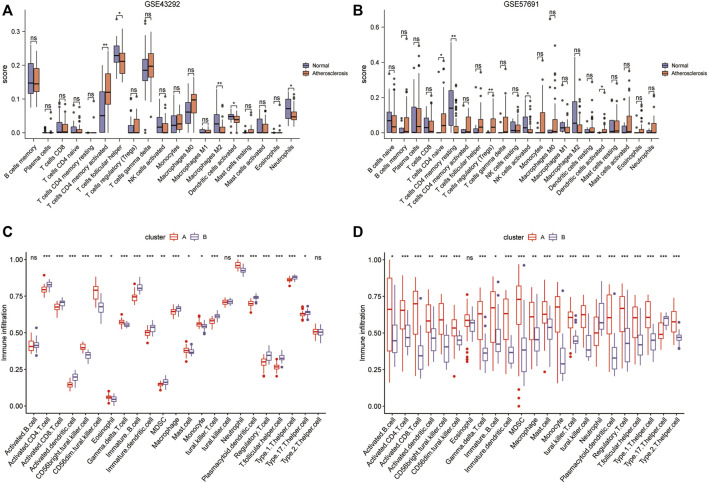
Construction of immune signature subtypes and analysis of immune cell infiltration assessment. **(A)** Box plots of differential immune cell infiltration between normal and atherosclerotic samples in the GSE43292 dataset by the CIBERSORT method; **(B)** box plots of immune cell infiltration in the GSE57691 dataset by the CIBERSORT method; **(C)** ssGSEA analysis box plots of the differential infiltration of immune cells in the 4-gene diagnostic model clustering subgroups among samples from the GSE43292 dataset; **(D)** ssGSEA analysis of the box plots of the differential infiltration of immune cells in the GSE57691 dataset.

The results of CIBERSORT deconvolution analysis of the two datasets revealed that the immune cells with significant differences in the GSE43292 dataset were CD4 memory activated T cells, follicular helper cells, M2 macrophages, activated dendritic cells, and neutrophils (Figure 12A). Immune cells with significant differences in the GSE57691 dataset were CD4 naïve T cells, CD4 memory resting T cells, regulatory T cells (Tregs), activated NK cells, and activated dendritic cells (Figure 12B). Subsequently, the ssGSEA results suggested that in the GSE43292 dataset, immune cell infiltration, based on the four-gene diagnostic model clustering of subgroups for oxidative stress-related AS, was significantly different in terms of activated CD4 T cells, activated CD8 T cells, activated dendritic cells, et al. (Figure 12C). Immune cells in the GSE57691 dataset showed significant differences in infiltration, based on the four-gene diagnostic model clustering of subgroups of oxidative stress-related AS, in terms of activated B cells, activated CD4 T cells, activated CD8 T cells, et al. (Figure 12D).

### Correlation analysis of the four genes in the oxidative stress-related AS diagnostic model

**FIGURE 13:**
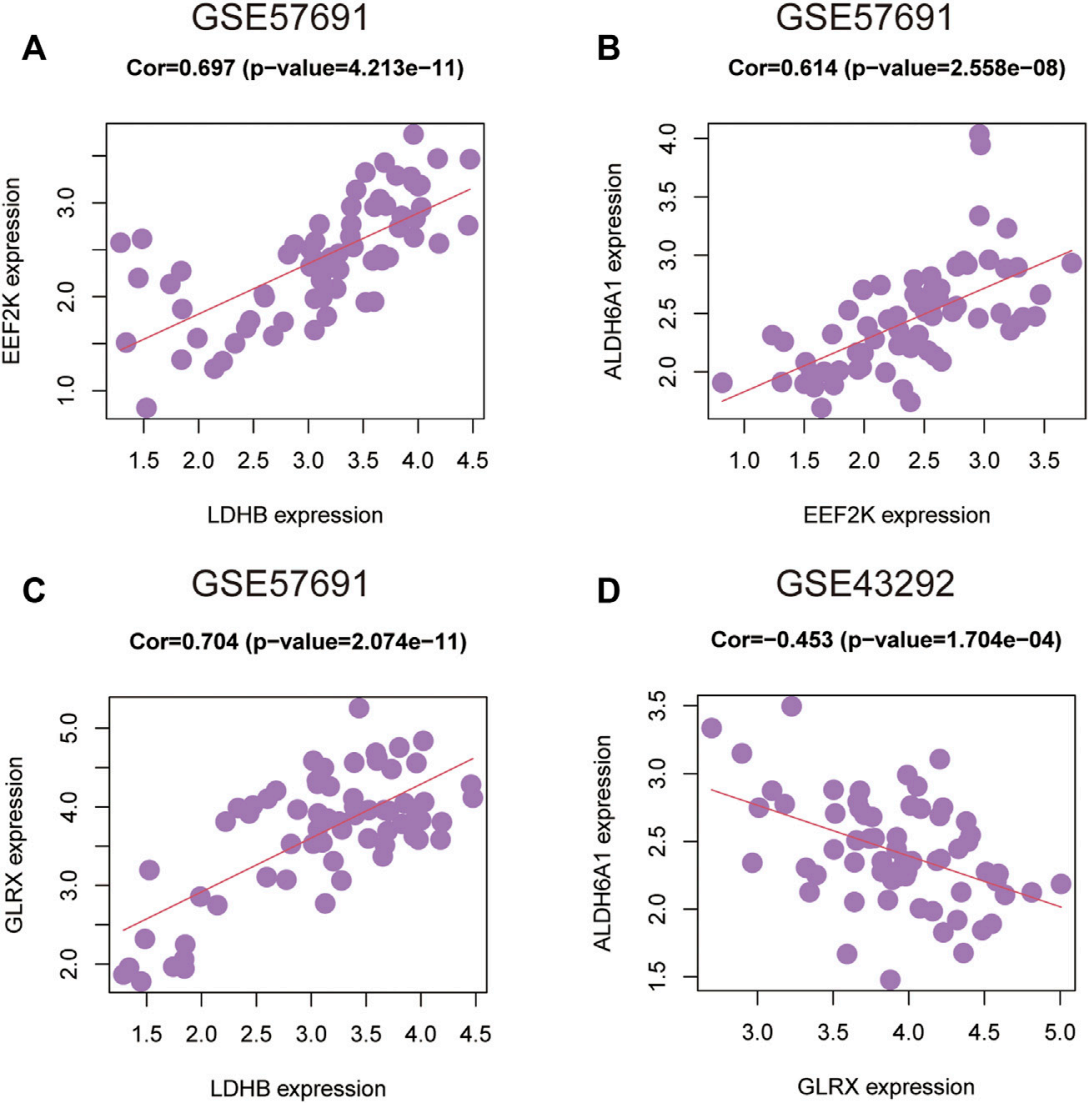
Correlation analysis between 4-gene diagnostic models of oxidative stress-related atherosclerosis in the GSE43292 and GSE57691 datasets **(A–C)** *ALDH6A1* and *EEF2K* (r = 0.614, *p* = 2.56E-08); *EEF2K* and *LDHB* (r = 0.697, *p* = 4.21E-11); *GLRX* and *LDHB* (r = 0.704, *p* = 2.07E-11) in the GSE57691 dataset **(D)** *ALDH6A1* and *GLRX* (r = -0.453, *p* = 0.00017) in the GSE43292 dataset.

To further understand the degree of correlation among the four genes in the diagnostic model of oxidative stress-related AS and the differences in correlations in the four genes between the GSE43292 and GSE57691 datasets, we presented the results in the form of scatter plots with correlation coefficients |R| > 0.6, which were statistically significant in the GSE57691 dataset. The results with a correlation coefficient |R| > 0.6 in the GSE57691 and GSE43292 dataset with statistical significance are presented in the form of scatter plots (Table 4). The results of *ALDH6A1* and *EEF2K* (r = 0.614, *p* = 2.56E-08), *EEF2K* and *LDHB* (r = 0.697, *p* = 4.21E-11), and *GLRX* and *LDHB* (r = 0.704, *p* = 2.07E-11) in the GSE57691 dataset were present in Figures 13A–C. The result of *DALDH6A1* and *GLRX* (r = −0.453, *p* = 0.00017) was showed in Figure 13D.

**TABLE 4:** Correlation analysis of the four genes in the diagnostic model

GSE57691			
Gene		Cor	adj.*p*
*ALDH6A1*	*EEF2K*	0.614	2.56E-08
*EEF2K*	*LDHB*	0.697	4.21E-11
*GLRX*	*LDHB*	0.704	2.07E-11
GSE43292			
*ALDH6A1*	*GLRX*	-0.453	0.00017

### Immune cell infiltration correlation analysis

**FIGURE 14:**
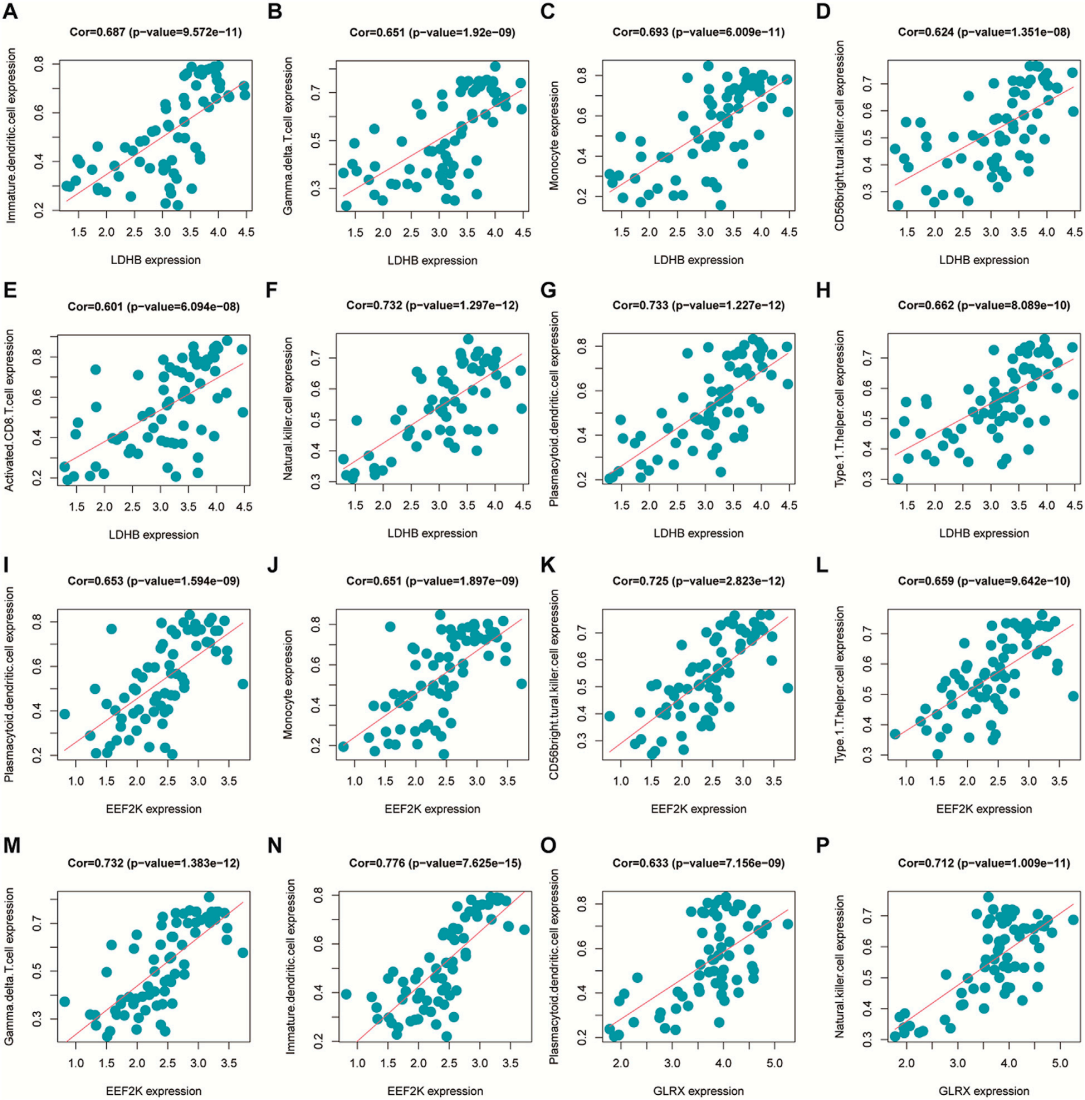
Scatter plot of the correlation between gene models and immune cell infiltration in the GSE57691 dataset.

**FIGURE 15:**
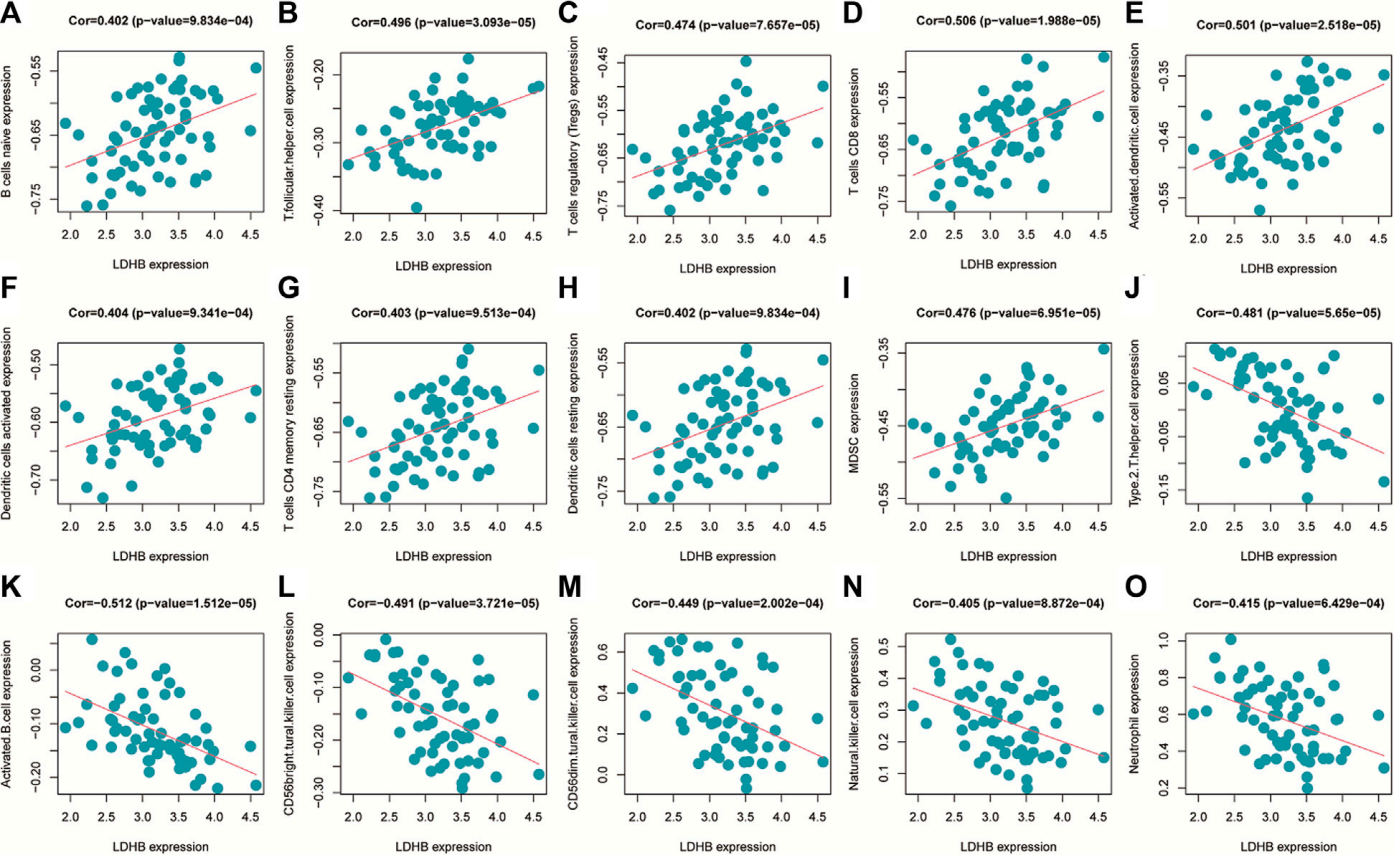
Scatter plot of the correlation between gene models and immune cell infiltration in the GSE43292 dataset.

The immune cell infiltration correlation analysis showed the results of our investigation of its immune microenvironment. The four-gene diagnostic models in the GSE57691 dataset correlated with infiltrating immune cells, including that *LDHB* was positively correlated with activated CD8 T cells (r = 0.601, *p* = 6.09E-08), CD56bright natural killer cells (r = 0.624, *p* = 1.35E-08), et al. *EEF2K* was positively correlated with monocytes (r = 0.651, *p* = 1.90E-09) and plasmacytoid dendritic cells (r = 0.653, *p* = 1.59E-09). *GLRX* was positively correlated with natural killer cells (r = 0.712, *p* = 1.01E-11) and plasmacytoid dendritic cells (r = 0.633, *p* = 7.16E-09) (Figure 14 and Table 5). In the other dataset GSE43292, the four genetic diagnostic markers correlated with many infiltrating immune cells, including *LDHB* with naïve B cells (r = 0.402, *p* = 0.000983), resting dendritic cells (r = 0.402, *p* = 0.000983) and CD4 memory resting T cells (r = 0.403, *p* = 0.000951) et al. (Figure 15 and Table 6).

**TABLE 5:** Correlation analysis of genetic models and immune cell infiltration in the GSE57691 dataset.

Gene	Immune cell	Cor	P-value
LDHB	Activated.CD8.T.cell	0.601	6.09E-08
	CD56bright.tural.killer.cell	0.624	1.35E-08
	Gamma.delta.T.cell	0.651	1.92E-09
	Immature.dendritic.cell	0.687	9.57E-11
	Monocyte	0.693	6.01E-11
	Natural.killer.cell	0.732	1.30E-12
	Plasmacytoid.dendritic.cell	0.733	1.23E-12
	Type.1.T.helper.cell	0.662	8.09E-10
	Type.17.T.helper.cell	-0.619	1.79E-08
GLRX	Natural.killer.cell	0.712	1.01E-11
	Plasmacytoid.dendritic.cell	0.633	7.16E-09
EEF2K	Monocyte	0.651	1.90E-09
	Plasmacytoid.dendritic.cell	0.653	1.59E-09
	Type.1.T.helper.cell	0.659	9.64E-10
	CD56bright.tural.killer.cell	0.725	2.82E-12
	Gamma.delta.T.cell	0.732	1.38E-12
	Immature.dendritic.cell	0.776	7.63E-15

**TABLE 6:** Correlation analysis of genetic models and immune cell infiltration in the GSE43292 dataset.

Gene	Immune cell	Cor	P-value
LDHB	Activated.B.cell	-0.512	1.51E-05
	CD56bright.tural.killer.cell	-0.491	3.72E-05
	Type.2.T.helper.cell	-0.481	5.65E-05
	CD56dim.tural.killer.cell	-0.449	0.0002
	Neutrophil	-0.415	0.000643
	Natural.killer.cell	-0.405	0.000887
	B cells naive	0.402	0.000983
	Dendritic cells resting	0.402	0.000983
	T cells CD4 memory resting	0.403	0.000951
	Dendritic cells activated	0.404	0.000934
	T cells regulatory (Tregs)	0.474	7.66E-05
	MDSC	0.476	6.95E-05
	T.follicular.helper.cell	0.496	3.09E-05
	Activated.dendritic.cell	0.501	2.52E-05
	T cells CD8	0.506	1.99E-05

## Discussion

AS, which includes coronary heart disease and stroke, is the leading cause of death worldwide (Iso, 2021). Coronary heart disease is estimated to affect 126 million people (1,655 per 100,000), accounting for approximately 1.72% of the global population (Khan et al., 2020). Mounting evidence links oxidative stress to the pathophysiology of AS, which is a chronic inflammatory disease characterized by lipid accumulation and swelling of the walls of medium and large arteries (Elahi et al., 2009; Yuan et al., 2019; Yin et al., 2022). However, the biological mechanisms underlying AS with oxidative stress remain unclear.

In this study, a bioinformatics analysis of the GSE57691 and GSE43292 databases was carried out, key genes were identified, and a four gene diagnostic model was constructed. The common DEGs were included after the comparison between the extracted module and the other dataset. Then, 17 hub genes based on the degree of connectivity were selected for further analysis. After LASSO logistic regression was performed and ROC curve was calculated, four DEGs (*ALDH6A1*, *EEF2K*, *GLRX*, and *LDHB*) with potential diagnostic value were identified as diagnostic markers. Furthermore, our data showed that these four genes were enriched in the following functional pathways: focal adhesion, cell-substrate adherens junction, cell-substrate junction on GO analysis; Alzheimer’s disease, Parkinson’s disease, prion disease pathways on KEGG analysis. The four-gene diagnostic model constructed in the present study included *ALDH6A1*, *EEF2K*, *GLRX*, and *LDHB*.

*ALDH6A1* is a member of the *ALDH* superfamily, which is highly involved in ROS production which increases dramatically in atherosclerosis (Ajoe et al., 2017; Yang et al., 2017). Studies (Calleja et al., 2021) have shown that aldehyde dehydrogenases (*ALDHs*) detoxify these aldehydes and protect cells from damage. And *ALDH6A1* is found in the liver, kidneys, heart, brain, and muscle (Kedishvili et al., 1992) and its deficiency results in developmental delay, which might play a role in the process of AS (Roe et al., 1998). In addition, in the present study, we found that *ALDH6A1* had relatively high diagnostic performance, with an AUC of 0.0.816 for AS in the GSE43292 analysis and an AUC of 0.845 for AS in the GSE57691 analysis.

*EEF2K* is also demonstrated to be responsible for atherosclerosis in one study (Beretta et al., 2020). Similarly, some tests had been performed to induce AS in mice with inactivating mutations in the *EEF2K* gene (Zhang et al., 2014). Usui et al. demonstrated that *EEF2K* controls the proliferation and migration of vascular smooth muscle cells, which mediate hypertension in tested rats *via* vascular inflammation (Usui et al., 2015). All these studies showed that *EEF2K* may be highly associated with inflammation and AS.

*GLRX* is another essential gene demonstrated in a previous study that its primary role is to control protein GSylation during oxidative stress (Burns et al., 2020). *GLRX* regulates GSylation of many proteins involved in signal transduction, such as phosphatases, kinases, and transcription factors, which contribute to the maintenance of cellular homeostasis. *GLRX* is associated with a wide variety of diseases such as Parkinson’s disease, non-alcoholic fatty liver disease, lung disease, and AS. There is evidence that *GLRX* expression increases in atherosclerotic coronary arteries (Okuda et al., 2001) and patients with type 2 diabetes (Du et al., 2014), suggesting that the upregulation of *GLRX* expression might contribute to inflammation and oxidative stress. Thus, *GLRX* plays an important role in oxidative stress associated AS.

*LDHB*, which catalyzes lactate conversion, is a glycolytic enzyme. *LDHB* deficiency plays an important role in diseases resulting from oxidative stress, as reported by Park et al. (Park et al., 2022). In addition, Wu et al. demonstrated that the knockout of *LDHB* significantly reduces H2O2 production, showing a direct correlation between *LDHB* and oxidative stress (Wu et al., 2021). Based on these findings, *LDHB* may be involved in the development of AS associated with oxidative stress.

Our study showed that the four-gene diagnostic model based on the combined expression of the four genes had good diagnostic performance based on GSE43292 and GSE57691 datasets. The AUC was 0.852 for GSE43292 and 0.967 for GSE57691. Therefore, this diagnostic model is likely to be used to evaluate patients with oxidative stress associated AS.

Besides construction of a diagnostic model, this study made an investigation the immune microenvironment of the four genes, considering that the immune microenvironment may further aggravate the progress of AS associated with oxidative stress. Based on our analysis by CIBERSORT and ssGSEA, it was found that the immune cells exhibiting significant differences in the two datasets included CD4 memory activated T cells, follicular helper cells, M2 macrophages, et al. The role and mechanism of T-cells, monocytes, and lymphocytes in AS are not clear, but the genes related to the immune system have become an potential target in the treating of AS. Emerging studies (Xu and Yang, 2020; Zhao et al., 2020) have revealed that immune cells participate in AS progression. Several experimental studies (Yin et al., 2021) have demonstrated that T cells play a key role in the immune response observed during atherogenesis. As cholesterol accumulates in the arteries and inflammation occurs, naïve CD4 T cells differentiate into adaptive cells, and the adaptive T cell response is induced. Atherogenesis increases memory CD4 T cell expansion and the generation of antigen-experienced T cells, which could be the result of re-exposure to antigens, and memory CD4 T cells will further elicit stronger and more sustained immune responses. Recent studies have demonstrated that immature dendritic cells are present in the endothelium of healthy arteries (Gao et al., 2016; Worbs et al., 2017). However, during the progression of atherosclerotic lesions, most dendritic cell populations appear to be activated and expand rapidly (Koltsova and Ley, 2011; Alberts-Grill et al., 2013). In the subendothelial space of the aorta, dendritic cells can accumulate lipids within the subendothelial space in the aorta and thus facilitate disease initiation and progression (Taghavie-Moghadam et al., 2014). Furthermore, activated dendritic cells are capable of producing proinflammatory molecules. As previous studies (Xu and Yang, 2020; Simões and Riley, 2022) have shown, CD8 T cells also contribute to the development of atherogenesis, as their numbers significantly increase as human lesions progress and become more vulnerable to ruptures.

This study has several limitations. First, the potential genes associated with oxidative stress identified in this study need further literature support and laboratory proof. Second, the genes associated with oxidative stress were derived from the GeneCards database, which is continuously being updated, and more genes need to be discovered. Third, to determine the diagnostic accuracy of the four-gene diagnostic model, larger sample sizes would be helpful for further external validation. Fourth, because AS is heterogeneous and clinical data are lacking, we were unable to evaluate the associations between risk indicators and the stratification of patients based on AS severity. Finally, experimental evidence, such as that obtained by real-time PCR, western blotting, and immunohistochemistry assays, is still required to fully understand the hub genes and underlying mechanisms of AS associated with oxidative stress.

## Conclusion

We identified 17 hub genes that were closely associated with oxidative stress in AS and constructed a four-gene (*ALDH6A1*, *EEF2K*, *GLRX*, and *LDHB*) diagnostic model with good accuracy. This model was also found to have good discriminatory efficacy for the immune cell infiltration microenvironment of AS. Overall, these findings provide valuable information and directions for future research into AS diagnosis and could aid in the discovery of the biological mechanisms underlying AS with oxidative stress.

## Data Availability

The datasets presented in this study can be found in online repositories. The names of the repository/repositories and accession number(s) can be found in the article/Supplementary Material.
