# Supplementary material for: Identification of oxidative stress-related genes and potential mechanisms in atherosclerosis
Source: Front Genet. 2023 Jan 4;13:998954. doi: 10.3389/fgene.2022.998954 (PMC9845256; doi:10.3389/fgene.2022.998954)
Supplement: Supplementary file 1 [file Table1.DOCX]

**Additional file.** List of Top30 GSEA enrichment analysis related to 4-gene diagnostic model

| ID | ES | NES | adj.p | q |
| --- | --- | --- | --- | --- |
| REACTOME_OLFACTORY_SIGNALING_PATHWAY | 0.552 | 2.973 | 0.039 | 0.029 |
| KEGG_OLFACTORY_TRANSDUCTION | 0.542 | 2.922 | 0.039 | 0.029 |
| REACTOME_G_ALPHA_S_SIGNALLING_EVENTS | 0.487 | 2.677 | 0.046 | 0.034 |
| REACTOME_KERATINIZATION | 0.483 | 2.378 | 0.032 | 0.024 |
| REACTOME_CLASS_C_3_METABOTROPIC_GLUTAMATE_PHEROMONE_RECEPTORS_ | 0.627 | 2.197 | 0.027 | 0.020 |
| KEGG_NEUROACTIVE_LIGAND_RECEPTOR_INTERACTION | 0.391 | 1.997 | 0.038 | 0.028 |
| WP_GPCRS_CLASS_A_RHODOPSINLIKE | 0.380 | 1.924 | 0.037 | 0.027 |
| REACTOME_DIGESTION_AND_ABSORPTION | 0.591 | 1.924 | 0.025 | 0.019 |
| WP_MAMMALIAN_DISORDER_OF_SEXUAL_DEVELOPMENT | 0.595 | 1.908 | 0.025 | 0.019 |
| REACTOME_PEPTIDE_HORMONE_BIOSYNTHESIS | 0.687 | 1.898 | 0.024 | 0.018 |
| REACTOME_DIGESTION | 0.605 | 1.874 | 0.025 | 0.018 |
| REACTOME_PHASE_0_RAPID_DEPOLARISATION | 0.502 | 1.865 | 0.039 | 0.029 |
| WP_SOMATIC_SEX_DETERMINATION | 0.673 | 1.857 | 0.024 | 0.018 |
| REACTOME_NA_CL_DEPENDENT_NEUROTRANSMITTER_TRANSPORTERS | 0.641 | 1.836 | 0.039 | 0.029 |
| REACTOME_VOLTAGE_GATED_POTASSIUM_CHANNELS | 0.506 | 1.834 | 0.039 | 0.029 |
| REACTOME_REGULATION_OF_GENE_EXPRESSION_IN_BETA_CELLS | 0.637 | 1.824 | 0.039 | 0.029 |
| REACTOME_AMINE_LIGAND_BINDING_RECEPTORS | 0.499 | 1.822 | 0.039 | 0.029 |
| REACTOME_HIGHLY_CALCIUM_PERMEABLE_POSTSYNAPTIC_NICOTINIC_ACETYLCHOLINE_RECEPTORS | 0.695 | 1.817 | 0.038 | 0.028 |
| PID_RHODOPSIN_PATHWAY | 0.567 | 1.810 | 0.050 | 0.037 |
| REACTOME_INTERACTION_BETWEEN_L1_AND_ANKYRINS | 0.514 | 1.800 | 0.039 | 0.029 |
| REACTOME_FORMATION_OF_THE_CORNIFIED_ENVELOPE | 0.391 | 1.779 | 0.031 | 0.023 |
| REACTOME_FERTILIZATION | 0.567 | 1.718 | 0.068 | 0.050 |
| WP_MONOAMINE_GPCRS | 0.497 | 1.712 | 0.039 | 0.029 |
| REACTOME_RETINOID_CYCLE_DISEASE_EVENTS | 0.653 | 1.706 | 0.066 | 0.049 |
| WP_NICOTINE_ACTIVITY_ON_DOPAMINERGIC_NEURONS | 0.550 | 1.704 | 0.067 | 0.050 |
| REACTOME_HORMONE_LIGAND_BINDING_RECEPTORS | 0.631 | 1.694 | 0.076 | 0.056 |
| REACTOME_PHASE_2_PLATEAU_PHASE | 0.519 | 1.690 | 0.061 | 0.045 |
| KEGG_TASTE_TRANSDUCTION | 0.441 | 1.665 | 0.064 | 0.047 |
| PID_CONE_PATHWAY | 0.534 | 1.655 | 0.092 | 0.068 |
| KEGG_MATURITY_ONSET_DIABETES_OF_THE_YOUNG | 0.546 | 1.654 | 0.077 | 0.057 |
| REACTOME_TRANSLATION | -0.652 | -2.968 | 0.019 | 0.014 |
| REACTOME_SRP_DEPENDENT_COTRANSLATIONAL_PROTEIN_TARGETING_TO_MEMBRANE | -0.689 | -2.818 | 0.019 | 0.014 |
| REACTOME_EUKARYOTIC_TRANSLATION_INITIATION | -0.671 | -2.766 | 0.019 | 0.014 |
| REACTOME_NONSENSE_MEDIATED_DECAY_NMD_ | -0.674 | -2.761 | 0.019 | 0.014 |
| REACTOME_SELENOAMINO_ACID_METABOLISM | -0.676 | -2.741 | 0.019 | 0.014 |
| REACTOME_RESPONSE_OF_EIF2AK4_GCN2_TO_AMINO_ACID_DEFICIENCY | -0.679 | -2.741 | 0.019 | 0.014 |
| REACTOME_EUKARYOTIC_TRANSLATION_ELONGATION | -0.690 | -2.720 | 0.019 | 0.014 |
| KEGG_RIBOSOME | -0.692 | -2.696 | 0.019 | 0.014 |
| REACTOME_INFLUENZA_INFECTION | -0.642 | -2.693 | 0.019 | 0.014 |
| WP_CYTOPLASMIC_RIBOSOMAL_PROTEINS | -0.688 | -2.676 | 0.019 | 0.014 |
| REACTOME_ACTIVATION_OF_THE_MRNA_UPON_BINDING_OF_THE_CAP_BINDING_COMPLEX_AND_EIFS_AND_SUBSEQUENT_BINDING_TO_43S | -0.724 | -2.661 | 0.019 | 0.014 |
| REACTOME_REGULATION_OF_EXPRESSION_OF_SLITS_AND_ROBOS | -0.606 | -2.624 | 0.019 | 0.014 |
| REACTOME_RRNA_PROCESSING | -0.603 | -2.609 | 0.019 | 0.014 |
| REACTOME_SIGNALING_BY_ROBO_RECEPTORS | -0.581 | -2.569 | 0.019 | 0.014 |
| REACTOME_REGULATION_OF_MRNA_STABILITY_BY_PROTEINS_THAT_BIND_AU_RICH_ELEMENTS | -0.652 | -2.504 | 0.019 | 0.014 |
| REACTOME_MITOCHONDRIAL_TRANSLATION | -0.634 | -2.479 | 0.019 | 0.014 |
| REACTOME_CELLULAR_RESPONSES_TO_EXTERNAL_STIMULI | -0.497 | -2.394 | 0.019 | 0.014 |
| REACTOME_AUF1_HNRNP_D0_BINDS_AND_DESTABILIZES_MRNA | -0.657 | -2.386 | 0.019 | 0.014 |
| KEGG_PARKINSONS_DISEASE | -0.603 | -2.364 | 0.019 | 0.014 |
| REACTOME_ANTIGEN_PROCESSING_CROSS_PRESENTATION | -0.597 | -2.361 | 0.019 | 0.014 |
| WP_PROTEASOME_DEGRADATION | -0.640 | -2.345 | 0.019 | 0.014 |
| REACTOME_THE_CITRIC_ACID_TCA_CYCLE_AND_RESPIRATORY_ELECTRON_TRANSPORT | -0.556 | -2.328 | 0.019 | 0.014 |
| WP_ELECTRON_TRANSPORT_CHAIN_OXPHOS_SYSTEM_IN_MITOCHONDRIA | -0.622 | -2.328 | 0.019 | 0.014 |
| REACTOME_RESPIRATORY_ELECTRON_TRANSPORT | -0.603 | -2.311 | 0.019 | 0.014 |
| REACTOME_UNFOLDED_PROTEIN_RESPONSE_UPR_ | -0.594 | -2.309 | 0.019 | 0.014 |
| KEGG_OXIDATIVE_PHOSPHORYLATION | -0.576 | -2.288 | 0.019 | 0.014 |
| REACTOME_HOST_INTERACTIONS_OF_HIV_FACTORS | -0.553 | -2.276 | 0.019 | 0.014 |
| REACTOME_TBC_RABGAPS | -0.670 | -2.254 | 0.019 | 0.014 |
| REACTOME_NEGATIVE_REGULATION_OF_NOTCH4_SIGNALING | -0.635 | -2.251 | 0.019 | 0.014 |
| REACTOME_MAPK6_MAPK4_SIGNALING | -0.582 | -2.245 | 0.019 | 0.014 |
